# Involving older adults in technology research and development discussions through dialogue cafés

**DOI:** 10.1186/s40900-021-00274-1

**Published:** 2021-05-10

**Authors:** Anne Lund, Torhild Holthe, Liv Halvorsrud, Dag Karterud, Adele Flakke Johannessen, Hilde Margrethe Lovett, Erik Thorstensen, Flávia Dias Casagrande, Evi Zouganeli, Reidun Norvoll, Ellen Marie Forsberg

**Affiliations:** 1 Faculty of Health Sciences, Department of Occupational Therapy, Prosthetics and Orthotics, Oslo Metropolitan University, PO Box 4, St. Olavs Plass, 0130 Oslo, Norway; 2 Department of Nursing and Health Promotion, Faculty of Health Sciences, Oslo Metropolitan University, PO Box 4, St. Olavs Plass, 0130 Oslo, Norway; 3 The Norwegian Board of Technology, Kongens gate 14, 0153 Oslo, Norway; 4 Oslo Metropolitan University, Work Research Institute, PO Box 4, St. Olavs Plass, 0130 Oslo, Norway; 5 Faculty of Technology, Art, and Design, Department of Mechanical, Electronics, and Chemical Engineering, Oslo Metropolitan University, PO Box 4, St. Olavs Plass, 0130 Oslo, Norway; 6 NORSUS Norwegian Institute for Sustainability Research, Stadion 4, 1671 Kråkerøy, Norway

**Keywords:** Assisted living residents, Assistive technology, User involvement, Dialogue cafés, Older adults

## Abstract

**Background:**

Citizen involvement is important for ensuring the relevance and quality of many research and innovation efforts. Literature shows that inadequate citizen involvement poses an obstacle during the research, development, and implementation of assistive technology. Previous studies have addressed the advantages and disadvantages of citizen engagement in health research and technology development, and there is concern about how to ensure valuable engagement to avoid situations where they don’t have influence. Frail older adults are often excluded from being active partners in research projects.

The overall objective of this commentary is to describe a case where dialogue cafés was used as a method for involving assisted living residents in technology discussions, elaborating on the following research question: In what ways are dialogue cafés useful for directing research and development and for engaging residents in assisted living facilities in assistive technology discussions?

**Method:**

Six dialogue cafés with assisted living residents as participants were carried out over a period of 3 years (2016–19). Reports that were written after each café by the group leaders and rapporteurs provide the material for the analyses in this paper.

**Results:**

This study demonstrates an example of facilitating user involvement where the participants felt useful by contributing to research and discussions on assistive technology and where this contribution in fact directed the research and development in the overall Assisted Living Project.

**Conclusion:**

This study demonstrated that dialogue cafés enable older residents at an assisted living facility to contribute with opinions about their needs and perspectives on assistive technologies. This negates the view of older adults as too frail to participate and demonstrates the importance of including and collaborating with older adults in research.

## Plain Language Summary

Assistive technology is used to provide older adults with the opportunity to preserve their quality of life and their ability to cope in everyday life live independently longer and enhance participation in everyday life. This commentary describes dialogue cafés as a method for involving residents in an assisted living facility in discussions about assistive technology. It elaborates on how dialogue cafés are useful for directing research and development and for engaging older adults in assistive technology discussions. Six dialogue cafés were held between 2016 and 2019 with residents (aged between 65 to 92) as participants in an assisted living facility in Norway. Reports that were written after each café by the group leaders and rapporteurs provide the material for the analyses in this paper. The study demonstrated that the engagement process was found to be positive by the participants in the dialogue café groups. It also succeeded in directing the research and technology development in the project. The dialogue cafés seemed to demonstrate a two-way learning process for the researchers and the participants.

## Background

The ageing of society has been declared as one of the grand future challenges of our times [1–3]. The challenges are related to escalating health care costs and increasing health disparities [4]. This is driving a renewed focus on developing health care services in other ways with the intention of reducing costs while at the same time enhancing seniors’ participation in everyday life [4]. Assistive technology is one important strategy for meeting the ageing society challenges, aiming to provide older adults with the opportunity to preserve their quality of life (seeing friends, enjoying hobbies, etc.) and their ability to cope in everyday life (cooking, paying bills, etc.), live independently longer, and also to reduce costs in health care services [5, 6]. Assistive technologies are new digital solutions that may include new, portable communication platforms in the community health services at home, tablets for communication in assisted living facilities, monitoring devices to be used at home, etc. (for a full list, see the literature review in Forsberg et al. 2020 [7]. Research and technology related to such a digital transformation, including changes at the organisational level within the health care and business sectors, and at the individual level [8], are therefore currently of high priority. However, some studies demonstrate a mismatch in priorities for research topics among patients, clinicians and the research communities [9, 10]. Moreover, involving citizens and stakeholders in the development process in an active way is currently argued to be an important driver of research and innovation [11] and may increase the relevance and quality of knowledge formation [12]. Citizen involvement increases empowerment and a sense of ownership of research among participants [11]. There is thus currently a growing recognition of the importance of involving users or stakeholders in research [4, 13]. Citizen involvement aiming for research to be useful for users and society at large, ultimately increases the societal support for investments in research and innovation.

With regard to studies of assistive technology more specifically, a similar observation is that inadequate citizen involvement poses an obstacle during the development, and implementation of technology [14–17]. Engaging older adults in technology development relevant to them is important to address end users’ requirements and to adapt the technology to users’ needs in order to succeed in technology uptake [18–20]. User involvement can also be important to evaluate the effectiveness and adoption of technology [21]. The level of willingness to install technological solutions at home is high if it enables and empowers end user to live at home [15]. Holroyd-Leduc et al. (2016) report on a literature review that addresses engaging frail older adults in research, in which they acknowledge the challenges, yet confirm that engaging this population is feasible. They state that ‘in a democratic society, citizen engagement is an ethical imperative which embraces the principles of inclusivity, mutual respect and co-design.’ (p. 13) [22]. However, though many studies have confirmed the advantages of citizen engagement in health research, there is also concern about how to ensure meaningful engagement and avoid that users have no influence [13, 23]. A scoping review demonstrates that frail adults (e.g. older adults with health problems) are rarely included in research [10]. Burrows et al. (2019) evaluated user involvement in an interdisciplinary technology research project and showed that user involvement adds value to many aspects of health research, but that there is a need to further consider how to engage users to become involved in interdisciplinary contexts [24]. Banner et al. (2019) explore some theoretical and conceptual considerations regarding user engagement in research and in integrated knowledge translation (IKT), and raise the need for further research regarding how researchers address power and diversity to enhance co-production to create impactful research [4].

In the health research and innovation field, different collaborative models of research involving end users, decisions makers, health care providers, policy makers, caregivers, patients, next of kin and citizens have been developed in recent decades [4]. Co-creation describes an interaction whereby actors create a mutually valued outcome, based on dialogue on risks and benefits, common access to information and resources, and transparency [25]. Co-creating interactions between end users, other non-academic stakeholders (peers or family) and academic researchers can ensure a good fit with end-user needs [26, 27]. Co-creation is believed to increase real world relevance across different disciplines where knowledge is created through joint knowledge production rather than through knowledge translation [28]. Co-creation is described as engagement by stakeholders in the process of mutual learning and in identifying solutions [29]. One widely accepted understanding of this type of involvement is research that is carried out ‘with’ or ‘by’ members of the public rather than ‘to’, ‘about’ or ‘for’ them [24]. In our study we share this intention of doing research ‘with’ residents in an assistive living facility. Despite the clear benefits of including older adults in research and technology development in the health field, an important question is the methodological issue of how to involve and engage older adults in questions of assistive technology and even artificial intelligence. These topics are considered by many to be highly technical and require high cognitive abilities to relate to intelligibly. Showing respect for older adults as a heterogeneous group with varying capabilities impels us to try to design ways of engagement that enable the participants’ to experience being heard and at the same time are useful for directing research and development in assistive technologies. In literature there is limited discussion of how the involvement process influences the research and development decisions and what the researchers learned [30]. In this commentary we will elaborate on and discuss an example of user involvement in an assistive technology research project (the Assisted Living Project (ALP)) with residents in an assisted living facility in Norway.

The transdisciplinary Assisted Living project (2015–2019) conducted research within information and communication technology -ICT, health science, social science and ethics. The overall aim of the project was to advance responsible research and innovation (RRI) in the field of assistive technologies. The project included technology development and research, an interview study conducted in the community health services at home in Oslo, focus group interviews with health care staff in Oslo, technology discussions and trials in an assisted living facility in Oslo (reported in this commentary), and research on the RRI dimensions of the project. The RRI framework applied in the project was a version developed in Wickson and Forsberg (2015) [31] and emphasised the importance of the following dimensions of responsibility in the research and innovation process:

1. A specific focus on addressing significant societal needs and challenges,
2. A research and development process that actively engages and responds to a range of stakeholders,
3. A concerted effort to anticipate potential problems, identify alternatives, and reflect on underlying values, and
4. A willingness from relevant actors to act and adapt according to 1–3. (see Forsberg and Thorstensen 2018) [32]

As part of addressing dimensions 2 to 4, we designed two involvement processes in the project. The Integrated Assessment group (also called the ProjectSTEP group) was an advisory group that included users, user-organizations, carers, professionals and researchers from quite different traditions, both within ethics, health care and technology development. This group met twice a year over 4 years to help the consortium reflect on the research and development process. The second involvement process is the one reported on in this commentary; where residents from the assisted living facility that took part in the project were invited to collaborate with a multidisciplinary research team consisting of partners in the fields of engineering, ethics, nursing, occupational therapy, sociology, technology assessment and technology development [33]. This collaboration consisted both of engaging a larger number of residents in dialogue cafés and a smaller number in technology trials in their apartments. This facility was selected as the residents there had for a period been provided with assistive technologies (e.g. computer tablets) and could therefore be assumed to relate easily to the topics of the project. The overall objective of this commentary article is to describe dialogue cafés as a method for involving assisted living residents in technology discussions, elaborating on the following research question: In what ways are dialogue cafés useful for directing research and development and for engaging older adults in assistive technology discussions?

As the methodology was specifically adapted to this target group, the experiences from the study are likely to be of particular interest to other researchers with similar ambitions of including and collaborating with older adults in technology research and development. For more information about the design of the project and results, see also Zouganeli et al.2017 [34]. Holthe et al. [16, 35, 36], Casagrande [7], Holthe [12] and Thorstensen [37].

## Methods

### Dialogue cafés

In ALP, dialogue cafés were conducted to engage residents in an assisted living facility in collaborating with the abovementioned multidisciplinary research team. The dialogue café design was inspired by well-known methods for user involvement, especially world cafés [38] and dialogue conferences [39]. The world café method was created by Juanita Brown and David Isaacs in the 1990s and follows the principle of good conversation, where all participants are given the chance to talk about things that matter to them [38]. The method follows two principles: first, people want to talk together about things that matter to them; and second, if they do, they could create collective power. The design is based on the assumption that people inherently possess the wisdom and creativity to confront even the most difficult challenges [38]. The world café method generates new ideas, enables joint decision-making on key strategic issues, discovers new ways of collaboration, reflects on the implications of complex issues and identifies specific step(s) for further exploration and implementation [38, 40]. The world café method is reported to be a valuable participatory, flexible method that can be used with community and health care stakeholders for research prioritisation with marginalised communities [41, 42]. Dialogue conferences are well-known from action research in work place studies, where the purpose is to create more democratic problem framing and problem solution across established groups (such as company management and workers). The democratic commitment of dialogue conferences translates well into the democratic commitments of an RRI approach, but a conference setting was deemed to be too large for this target audience.

Inspired by both world cafés and dialogue conferences, we therefore found ‘dialogue cafés’ to be in line with the aims related to user involvement in RRI , and, more specifically, with the intention to engage residents in an assisted living facility in assistive technology discussions.

### The dialogue café method in the assisted living project

In order to include and engage the residents of the assisted living facility in the dialogue cafés, we carefully planned each café meeting, in line with the recommendations of Kennedy and ter Meulen [43]. Dialogue cafés were selected for user involvement from the beginning of the Assisted Living Project. However, the number of dialogue cafés was not decided. The specific content of each dialogue café was defined as the project proceeded, and the next café was initiated when more knowledge of the residents’ needs and opinions was required for the project. This resulted in six dialogue cafés being conducted in the assisted living facility. The cafés was led by the researcher who were either group-leader or rapporteur.

Prior to each of the six dialogue cafés, the group leaders and rapporteurs developed written instructions describing the aims and intended content for the café, including tasks and questions to be discussed. Two of the researchers led the work of planning each café in dialogue with the whole research group, who filled the roles of group leaders and rapporteurs. A few days before each dialogue café, all the researchers met and performed a ‘dry-run café’ giving the group leaders and rapporteurs a common understanding of the content, aims and questions asked and how to follow the method. After the dry-runs were performed, adaptations were made and final instructions agreed upon. On the day of the café, the researchers met one hour ahead to agree on final details. The researchers held a one-hour summing-up meeting after each café to evaluate the content and the process and reported in pre-prepared templates their impressions and opinions of the discussions and their own roles, challenges and successes during the cafés. Each café lasted for two hours. Café tables for three to six residents, one group leader and one rapporteur were organised in the cafeteria at the assisted living facility. Coffee, tea and snacks were offered at the beginning of each meeting.

**Fig. 1:**
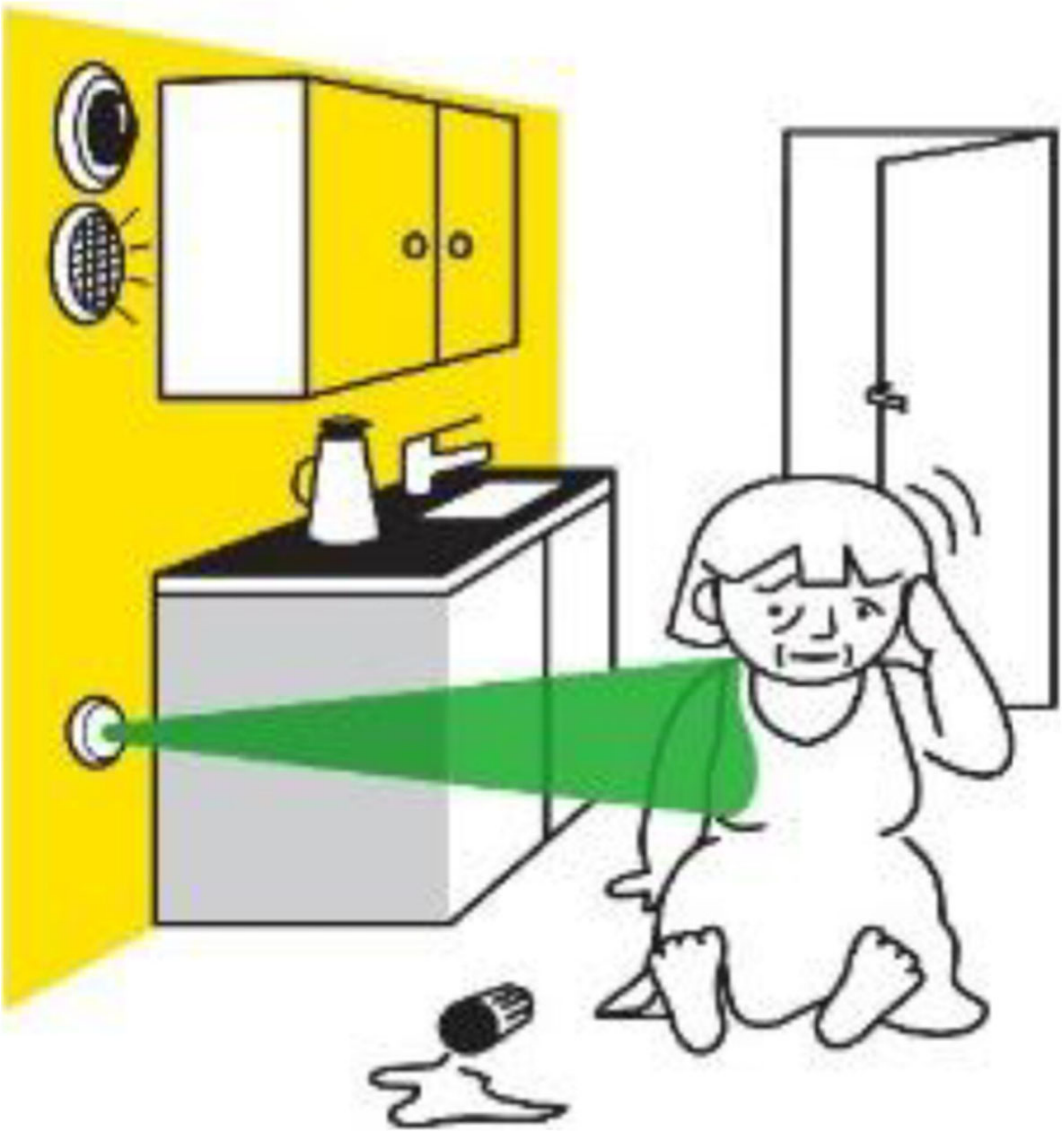
Nora has fallen and a video camera has registered it. Illustration from dialogue café no. 2

Each table had a group leader and a rapporteur. Paper, stickers, pens, name labels and consent sheets were provided. After written consent was obtained from the participants, each café started with a plenary presentation by a facilitator. Illustrations with two fictional cartoon figures called ‘Helmer’ (male, 85 years old) and ‘Nora’ (female, 77 years old) were used at each café to demonstrate how the technology could contribute in everyday life and to facilitate the discussions (see example in Fig. 1).

By using a combination of plenary sessions and group discussions, as well as illustrations and technology props, we aimed to avoid wearing out the participants.

The facilitator was responsible for all the groups keeping time and following the plan. The group leaders facilitated the discussions at their respective tables, assisted by the rapporteurs, in line with the recommendations of the Engage 2020 project [40] and MacFarlane et al. [41].

### Recruitment and ethics

The assisted living facility included in the study consists of apartments that are physically adapted to older adults who have challenges in managing their everyday lives though they do not yet need a nursing home placement. In order to live in the facility, the residents must to some extent be able to live independently. They may have functional impairments such as: a) physical disabilities i.e. impaired mobility, b) chronic and long-lasting and severe mental health problems or c) decreased function due to high age. Individuals with significant cognitive impairments or with chronic and long-lasting high degrees of mental problems do not qualify for renting an apartment in the assisted living facility [44]. Nevertheless, some residents develop cognitive impairments after moving in, or may not have manifested such symptoms at the time when their application was submitted, so at any time there is a diversity in the cognitive functioning of the residents. The assisted living facility in the study included an activity centre, a cafeteria and a reception open from 8 AM to 4 PM with a housekeeper available during the day. The housekeeper team knew all the residents well and contributed with information about the project and motivated residents for participation.

We proceeded one step at a time in order to invite, recruit and retain participants in the study. Introductory presentations and discussions with the leaders and housekeepers in the assisted living facility were used to anchor the project. The residents from all 53 apartments were thereafter invited to a presentation of the project at one of the regular ‘house meetings’, at which all the project’s researchers, as well as its aims and objectives, were presented.

The researchers visited the assisted living facility approximately two or three times a week in the beginning, to get acquainted with the residents and to repeat information about the project. The housekeeper played an important role in recruiting residents to take part in the interviews and in the dialogue cafés. In October 2016 all the residents were invited to the first dialogue café (DC1, see Table 1 for more information). They received an invitation in their private mailbox and an open letter was posted on a bulletin board in the cafeteria. All participants were asked to sign an informed consent form before participating in each dialogue café. We sought to write this in plain language while still conveying sufficient detail for making an informed choice about participation. The information was also provided orally by the project researchers. With the housekeepers’ help, the residents’ competence to give consent and ability to participate in the research project was continuously assessed throughout the project, in line with the recommendations of Kennedy and ter Meulen [43].

**Table 1:** Overview of participants at each café

DC number	Total number of participants	Men/women
DC1	13	4/9
DC2	13	4/9
DC3	14	2/12
DC4	11	2/9
DC5	12	3/9
	12	3/9

The privacy and data protection aspects of the project were assessed and approved by the Norwegian Centre for Research Data (NSD) on 16 March 2016, application number 47996.

### Data

The main material for the analyses comprises the six written reports from each café. Each report includes the templates completed by each group’s leader and rapporteur, as well as a general summary of each café. The reports also included the researchers’ individual reflections on the aims for and experiences of the dialogue cafés.

### Process of analyses

The written material was analysed using a qualitative content analyses [45]. The analyses started off using an explorative approach close to the written material. First, all the researchers read the reports from the six dialogue cafés and tried to use a ‘bird’s eye’ perspective before meeting face to face to share and discuss their perspectives. Second, two researchers reviewed the reports more systematically and identified areas and added keyword to the text [45]. Themes emerged through the work that matched our research interests and we chose to further analyse the material in line with clearly formulated research questions. We thus analysed the reports focusing on how the participants seemed to create engagement in the dialogue café discussions and how the researchers used the participants’ valuable comments for directing the research. In other words, we sought to assess both the participants’ and the researchers’ experiences with the process.

Below we also include information on the content of the discussions as it demonstrates how the cafés helped in the development of the project, which is essential in light of the project’s RRI perspective and the second research question.

## Results

First, we will present an overview of the participants, followed by descriptions of the dialogue cafés. We will then present extracts from the reports on the participants’ experiences of the dialogue cafés. Finally, we will present reflections on the usefulness of the DCs for directing the research and development in the project.

### Overview of the participants

Each of the six dialogue cafés was attended by between 11 and 14 residents ranging in age from 65 to 92. Few men participated, due to the fact that the residents of the assisted living facility were mostly women.

The six dialogue cafés built on each other as described in the subsequent section. Each café started with a reminder of what was discussed at the previous café(s), showing the logical progression of the dialogue cafés, allowing for new participants to understand the purpose of the café in a larger context, and also reminding recurring participants about what they had discussed earlier. The cafés ended with a summary validated by the participants. Thirteen of the participants attended all the cafés. Non-attendance was generally related to health issues (i.e. not feeling well at the time of the café or being temporarily in the hospital). The groups were organised once the participants had arrived, as we could not know in advance, who would show up. The principles for setting up the group were that each group should not have less than three participants and there should be a variety in gender, if possible. In order to avoid collecting more personal information than necessary, we did not record the names of the group participants in the reports. The group participation therefore changed from café to café and validation of the reports from the groups by the participants was not possible.

### The aims, content and main results of the dialogue cafés

#### DC1

The aim of this DC was to identify the participants’ perceived needs related to the most important challenges in their everyday life. The DC resulted in the following list of challenges: i) The fear of falling, being injured and not getting help; ii) Being outdoors and having access to fresh air, being safe when out of the house; iii) Ability to orient oneself at night. Dark environments and possibly impaired vision may influence navigation/orientation at night; iv) ‘Button-phobia’ where technology can be difficult to use due to small buttons and unfamiliar interfaces, passwords, and codes; v) Social contact with friends and relatives due to e.g. immobility; vi) Safety at home. This is multifaceted and relates to the fear of not getting help promptly when required; vii) Sleeping sufficiently and well. Challenges include problems with falling asleep, waking up frequently during the night, and/or waking up too early in the morning; viii) Self-sufficiency and autonomy. Even if the residents do feel relatively autonomous and self-sufficient, their daily routines need to conform to those of others such as the schedules of the community health services at home (e.g.nurses, physiotherapists, occupational therapists or others). In addition, the following priorities became apparent from a simple polling: i. The high importance/priority of being independent, self-sufficient, and in control of one’s life. Most of the residents were also wary of troubling their family and friends; ii. The wish to remain active and a fear that relying on help from others or from a system may cause deterioration of their cognitive ability; iii. A willingness to trade privacy for better safety.

#### DC2

The aim of this DC was to present some technological solutions related to the participants’ perceived needs and challenges discussed at DC1. The researchers invited discussion of four illustrated stories with Nora and Helmer. These were:

i. The bathroom and bedside lights switch on automatically when Helmer gets out of bed at night. Many participants found this very useful.

ii. An alarm is activated when Nora falls in the kitchen. Her daughter gets a message, and they can talk via loudspeakers while waiting for assistance to come. The camera can also be turned on. This solution raised discussions on the need for not ‘disturbing’ next-of-kin. However, they reported that this might be a good solution.

iii. A voice message reminds Nora not to forget her wallet when she is about to leave her apartment. The participants did not find this solution useful.

iv. While sitting in his apartment, Helmer can use his tablet to find out who is in the cafeteria so that he can consider joining them. The participants did not embrace this solution; they would prefer to go and visit the cafeteria and meet with those who were present.

#### DC3

The aim of this DC was to acquaint the participants with available low-technology assistive solutions through hands-on experience with prototypes, followed by an invitation to take part in a field trial of environmental sensors. The commercial technology partner made mock-up versions of the technology so that the residents could test the solutions and see for themselves how technology can work. They were eager to learn how the prototypes worked. This contributed to fruitful discussions concerning ethical issues, usefulness and user interface of technology. They also showed interest in how the solutions could be useful in their own apartments.

#### DC4

The aim of this DC was twofold. First, to get feedback on the perceived usefulness of a fall detection system based on a demonstration of the movement detection system Microsoft Kinect. The participants found this useful, and ethical issues were discussed concerning whether the system could be installed in the bathroom. While the researchers had assumed that the participants would object to having cameras installed in the bathroom out of privacy concerns, the participants instead emphasised their fear of falling in the bathroom. The second aim was to build on the findings from DC3 and invite the participants to test two different solutions in their homes: i) a physical switch by the bedside enabling the participants to turn the bathroom light on and off remotely (e.g. when getting out of bed during the night), and ii) an automatic vocal message from a loudspeaker when leaving the apartment asking whether the stove or coffee machine is on, or whether the balcony door or fridge door is open. Eight residents consented to participate in trying out such a smart-home system in their apartments as a field trial. They tried out the technology for 7 months and had a phone number to the researchers who gave support when they needed. The housekeeper also contacted the researchers when needed. These findings are presented in other papers [35, 36].

#### DC5

The aim of this DC was threefold. First, to demonstrate and explain machine learning related to another, smarter fall detection system called RoomMate. Second, to get feedback on four potential future functions that could be provided using machine learning. The latter goal was facilitated with illustrations of the by then well-known Nora and Helmer:

i. Fall risk assessment. Using RoomMate, it is possible to deduce Helmer’s motion pattern and whether he is unsteady or his balance has deteriorated. The system could notify Helmer himself, the host, or Helmer’s family.
ii. Hazardous situation alert. Using computer vision, it is possible to register when Nora is in bed and has forgotten to switch off the stove. A smart system should be able to adapt to Nora’s routines and turn the stove off if it is forgotten and there is a risk of overheating.
iii. Comfort automation. The light in the kitchen and the coffee machine are automatically switched off when Nora goes to bed at night. The lights in the apartment are automatically controlled to give her better light when she gets up at night. The bedside lamp turns on when she gets up at night. The bathroom lights switch on if she intends to go to the bathroom and turn off a sufficient time after she leaves the bathroom. The bedside lamp turns off when she finishes reading and turns over to sleep.
iv. Assistance with switching on the TV. Using computer vision based on the data from RoomMate, the system understands that Nora struggles to switch on the TV, and switches it on automatically at the channel Nora usually watches at a certain time on a certain day.

The third aim was to recruit participants for a further field trial of a smart home system in their apartments, with 13–17 sensors as well as two RoomMate fall detectors, one in the living room and one in the bedroom. Eight residents were recruited to the trial. The results from this study are presented in another paper [46].

#### DC6

The aim was to close the project, thank all the participants for their participation and let them express their opinions on participating in the project. They were eager to share that it had been very interesting and that they felt they had learned more about technology. They reported being satisfied with their involvement in the project. Please see Table 2 for an overview of the cafés.

**Table 2:** Overview of the six DCs

DC number/ date	Overall aim	Purpose	Content/method	Preparations
DC1 26.10.16	Explore the participants’ needs.	Understand the needs of the participants and especially identify their reported challenges in daily life. Enable the researchers to propose solutions to the participants reported needs.	Presentation of cartoon illustrations of Helmer and Nora in three examples of challenging situations to encourage discussions around needs and challenges in everyday life: i) an ordinary day at home ii) out and about iii) an ordinary night at home.	The research team developed the illustrations of Helmer and Nora and performed a dry-run café. Written instructions were developed.
DC2 14.12.16	Dissemination of the potential of future solutions via examples.	Show examples on how automation and smart-home technology can be helpful in daily life. Discuss four examples of technology to get feedback from the participants on the usefulness and acceptability of these solutions.	Four cartoons were presented: i. the bathroom and bedside light switch on automatically when Helmer gets out of bed at night. ii. An alarm is activated when Nora falls down in the kitchen. Her daughter gets a message, and they can have a talk via loudspeakers while waiting for assistance to come. The camera can also be turned on (See Fig. 1). iii. A voice message reminds Nora not to forget her wallet when she is about to leave her apartment without it. iv. Helmer in his apartment can get information on his tablet as to who is in the cafeteria so that he can consider to joining them.	The needs were discussed in the team, and several technology solutions that were assessed as relevant for the project as well as possible to realize within the project’s duration, were mapped to these needs. A possible smart-home sensor system was proposed to the project team and evaluated. Dry-run.
DC3 6.4.17	Test and evaluate low-tech existing solutions.	Understand the participants views aimed at an aquaintance with available low-technology assistive solutions by hands-on experiences with prototypes.	Demonstration of and testing simple available technological solutions: i. Helmer has a portable light switch that turns on/off the light that shows the way to the bathroom. The light can also be controlled by a tablet. ii. Nora pushes a button on the wall or tablet before leaving the apartment and a voice tells her if the terrace door is open or the fridge is open or the stove is on. iii. Nora demonstrates that by pressing one button on the tablet her favorite TV channel is switched on.	Map available low-tech solutions to a selection of needs. Make mock-up versions og solutions. Dry-run.
DC4 31.5.17	To get feedback on a fall detection system. Recruitment of participants for a field trial of existing technology.	To get feedback on the usability and applicability of a fall detecting module and service based on Microsoft Kinect. Invitation to test simple technology.	Fall detection: - demonstration of a fall detection system using Microsoft Kinect. - presentation of the fall detection and alert cartoon with Nora (reuse of cartoon illustration in DC2). Proposed system to install: i. physical switch by the bedside to turn the bathroom lamp on and off remotely, when standing up from bed at night. ii. vocal message when leaving the apartment if the stove or coffee machine is on, or the balcony door or fridge is open. The residents were invited to try these solutions at home for four weeks and give their opinion on usability and acceptability, as well as on how we could improve the solutions. Eight residents consented to take part in the trial, and one of them, Hilda, consented to participate in this feasibility study. The residents could choose which functionalities they wanted installed in their home.	Define solutions that are expected to have value for the participants based on the info from the previous cafés. In order to both get feedback on user experience, and in exchange for their providing data for further studies. Dry-run.
DC5 11.1.18	Recruitment of participants for a field trial to provide data for Machine Learning studies	Explain machine learning and demonstrate RoomMate as fall detection system and alert service. To get feedback on three examples of future functions that could be provided using machine learning. Invitation to participate in a trial of the RoomMate as fall detection and alert service as well as allow data collection for the development of future support functions.	Four cartoons were presented on future functions: i. Fall risk assessment: using RoomMate it is possible to deduce Helmer’s motion pattern and whether he is unsteady or his balance has deteriorated. The system can notify Helmer himself, the host, or Helmer’s family. ii. Hazardous situation alert: Using computer vision it is possible to register that Nora is in bed and has forgotten to switch off the stove. A ‘smart’ system should be able to adapt to Nora’s routines and turn the stove off if it is forgotten and there is a danger for overheating. iii. Comfort automation: the light in the kitchen and the coffee machine are automatically switched off when Nora goes to bed at night. The lights in the apartment are automatically controlled to give her better light when she gets up at night: The bedside lamp turns on when she gets up at night and the bathroom lights switch on if she intends to go to the bathroom; and turn off sufficient time after she has left the bathroom. The bedside lamp turns off when she finishes reading and turn round to sleep.. iv. Assistance – switching-on the TV: using computer vision based on the data from RoomMate, the system understands that Nora struggles to switch on the TV and switches it on automatically at the channel Nora usually watches on this day and time. The residents were recruited to get installed a smart-home system with 17 sensors, as well as two RoomMate fall detectors, one in the living room and one in the bedroom.	Define solutions that are expected to have value for the participants based on the info from the previous cafés. In order to both get feedback on user experience, and in exchange for their providing data for further studies. Meeting between researchers and one resident for discussing how to present machine learning in an understandable way. Dry-run.
DC6	Closing the project	Thank all the participants for their participation.	Discussed their opinions on their participating in the project.	

### The participants’ experiences from the dialogue cafés as reported by the research team

In general, the researchers reported that the groups seemed positive about the process. The group leader at the first café reported: ‘The atmosphere in the workshop was good, and the conversations at each table were positive.’ (DC1 report, p. 1). Another group leader reported that a participant expressed a similar positive attitude: ‘I enjoyed this café ... very good discussions and I felt that I was included.’ (DC1). A participant at DC2 reported: ‘This is interesting and intellectually stimulating.’ (DC2).

Group leaders used different strategies to achieve the good dialogue climate: ‘I tried to use humour and at the same time I tried to be open and curious and wanted to include all comments. I think this worked well.’ (DC1 report, p. 7). Some group leaders found it challenging to meet the expectations of the participants. One participant said at the first DC: ‘I expected we would discuss technology, as written in the invitation letter.’ The group leader then explained that the project wanted to start by discussing user needs or challenges in everyday life and then proceed to discuss technological solutions. Another group leader reported ‘Sometimes I wanted to give information about technology; however, I managed to keep quiet.’

One might suspect that some of the participants’ enthusiasm was due to the pleasant social situation. However, it appeared that their active contributions also felt empowering and created meaning. One report highlighted that one of the motivations for the residents was to be of benefit for others, exemplified by one group leader as follows: ‘They know they are helping us for future generations of older adults, which I believe is their own motivation.’ (DC2). This is further addressed in Holthe et al. 2020 [36].

Though the group discussions generally were successful, the leaders repeatedly found it challenging to manage the group process because they did not know the participants individually, and some participants appeared at times to be struggling to follow the conversation (DC2). Perhaps due to the careful design of the café, there seemed not to be significant problems of fatigue or decrease in engagement in the groups over the two hours.

### Usefulness for directing research

For the researchers it was important that participants properly understood the topics to be discussed. From the reports we can glean that this was largely the case, at least with active support from the leader. The leader of DC3 reported that ‘even though many of the residents seemed to like the solutions and gave valuable feedback about them, some needed time to understand them.’ (DC3). However, some challenges to the technological solution were revealed; ‘For example, one man didn’t want to have the remote light switch on the computer tablet because then he would need to have both the tablet computer and the external light switch at the bedside during night’ (DC3 report p.7). This lack of understanding would potentially decrease the value of the input to the research, but was not widespread in the groups. Regarding the cafes’ function in directing research, it is important to understand how the researchers experienced the process. User engagement needs to be experienced as relevant and useful for the researchers and innovators; otherwise it will not have an impact on the research and development. The dialogue cafés appeared to have such impact. A research member reported: (DC5) ‘The DCs were the most rewarding part of this interdisciplinary project for me personally, and where I learnt a lot … Meeting and discussing with the participants increased my understanding of their needs and opened my perspectives.’ Another fact demonstrating the usefulness of the contributions in the project was that we made changes after each café.

Overall, as demonstrated in the section above on the aims, content and main results of the dialogue cafés and in Table 2, the whole technology development process in the project was guided by input from the cafés.

## Discussion

We posed the following question: In what ways are dialogue cafés useful for directing research and development and for engaging older adults in assistive technology discussions? From the results, we conclude that the dialogue cafés served the purpose we intended in the project. The participants did indeed discuss the topics outlined for each café, which informed further research and technology development in the project related to the choice of solutions to develop and test in practice. We gained important insight into their needs, their daily challenges, their concerns about technology and their experiences in using the technology. The findings also demonstrate that the participants felt good about taking part in the cafés. Eleven of the initial group participants attending the first DC continued to participate in all the cafés. Several of them also wanted to try out technologies in their apartments, which is described in another paper [36]. Our findings demonstrate that the cafés can be seen as a valuable participatory, flexible method that can be used with marginalised communities [41], as assisted living residents are sometimes considered. From the reports of the leaders and rapporteurs, we believe that at least the following factors contributed to the success of the cafés:

- Presentation formats adapted to the participants: Using illustrations, presenters speaking slowly and clearly, information being repeated by the presenter in plenary and by the group leaders.
- Group dialogue formats adapted to the participants: The small size of the groups, the short duration of the discussion (including a break in the middle), informal and comfortable café setting with coffee and snacks, familiar location and familiar participants in the groups (they were all neighbours).
- Meaningful engagement through a mutual two-way learning process between the researchers and the participants.

These findings are supported by a study on public involvement in research [31]. The dialogue cafés demonstrate a good example of facilitating user involvement in that the participant felt useful by contributing to research that can help other people. This is also supported in another user involvement study [13]. The DCs can be seen as a process of engaging participants in ongoing dialogue using a variety of instruments related to mutual learning. Such a process does not need to be standardised [30], but can be tailor-made with a range of involvement techniques, such as the illustrations of Nora and Helmer.

### The illustrations of Nora and Helmer

The illustrations of Nora and Helmer were chosen to facilitate group discussions aimed at presenting and discussing technological solutions. The illustrations were also intended to create distance for reflection by not directly relating to the participants themselves. However, the experiences of using the stories of Nora and Helmer varied. Some of the participants perceived the illustrations to demonstrate two frail adults with whom they could not identify. Others expressed that the illustrations of Nora and Helmer were easy to discuss. One group leader reported that the stories of Nora and Helmer were not commented upon: ‘The residents preferred to talk about themselves instead.’ (DC1 report, p. 4). However, in DC2 one group leader reported that everybody had viewpoints on the illustrations and that Nora and Helmer contributed to opening discussions. Overall, the experience of the project group, in line with Crowe et al. [47], was that using the illustrations of Nora and Helmer facilitated reflections and mutual learning between researchers and participants.

### The engagement with technology issues in the cafés

The participants expressed that they enjoyed the coffee and cakes, and the social interaction. Despite the fact that several of the participants initially expressed little interest in technology, they appreciated being a part of the project and being included in discussions about future technology options. This negates the view of older adults as frail and unable to participate which may reinforce exclusions from discussions of technologies affecting their everyday life [36].

We cannot know how deeply the participants understood the technical features of the technologies they discussed, but this is not necessarily crucial, neither for their experience of being engaged nor for our use of their input. From the discussions, it was apparent that they understood the features of the technologies that were important to them (intrusion in daily life, safety, privacy, etc.). The groups occasionally strayed off the topic, but the group leaders helped to focus the discussions. We would emphasise that neither the depth of technical understanding nor the occasional distractions should be seen as major problems in engagement activities with older adults. Indeed, from our experience with conducting engagement activities this happens with most groups in such activities, even with groups consisting only of younger participants with no cognitive impairments.

### The co-creation in the project

This project had aspects of co-creation. Decisions about the technology development in the project was influenced by the input from the dialogue cafés. However, this input was used within limits of the actual technology competence and other resources (e.g. financial resources) available in the project. The design and implementation of the dialogue café approach and each specific café was primarily based on the reflections of the researchers based on the learning process throughout the project, and not co-created with the residents. An important input to the design was the thorough review of engaging frail older adults in research conducted by Kennedy and Ter Meulen [43]. The plan for the dialogue cafés was discussed with the ProjectSTEP group, which included, among others, a representative for the Norwegian Dementia Association (who had been next-of-kin to a woman with dementia) and a representative from the assisted living facility. In that sense, there was a certain co-creation of the cafés. However, the residents were not included in the ProjectSTEP group. There are several reasons for this. The most prominent was that such involvement would require full cognitive abilities. While there were several residents with excellent cognitive abilities, we were very careful not to create an ‘A team’ and a ‘B team’, where one group was privileged with more involvement and rights. Moreover, those not selected to be involved in the planning and implementation (for instance leading a café table) could easily feel stigmatised. This was especially important to avoid in a tightly knit community as this assisted living facility was where everybody knows each other and information flows freely. Moreover, as we wanted to be sure that the groups were facilitated in a way respecting the different abilities and characteristics of the participants, we made sure only certified health personnel of the research team led the groups. We could not have assured this if group leadership was left to the residents. In conclusion, we would say that the cafés were more than simply consultation, but not fully-fledged co-creation.

### Methodological discussions

The project was about assistive technology for and with older adults in an assisted living facility. The cognitive abilities of the residents varied widely. Thus, in collaboration with the housekeeper, we excluded residents who were assessed to be incapable of giving informed consent to participate. Everyone else was allowed to participate; to do otherwise might have induced social stigma. This means that there was a quite varied range of participants at the cafés, some with their cognitive abilities fully intact and some with reduced abilities. The group leaders were all health care personnel and were therefore aware of this situation. They tried to include all the residents in the group and to avoid some residents dominating over the others. This was done primarily by active facilitation of the group discussion, making sure everyone was involved. We also changed the group composition in the course of the six cafés in order to prevent persistent group dynamics from affecting individuals who were cognitively weaker. Another methodological issue is that the reports were written by the research team itself. Though we tried to give a neutral assessment in the reports, we wanted the cafés to be a success and worked hard to create a positive dialogical atmosphere. This might have skewed our reports slightly in a positive direction. Although we include direct quotes from participants in the reports, we may have subconsciously neglected or failed to report evidence indicating that the discussions did not work so well (though we have no reason to suspect this).

As is apparent from the descriptions of the progression of the dialogue cafés above, each café started with an introduction to the topic. This provided the project team with an opportunity to frame the discussion. Such framing was indeed necessary in order to have a structured discussion, but a potential framing effect must be acknowledged. The project researchers also took care to explain the technological solutions in a user-friendly way, which to a certain extent meant simplification. This was particularly the case when explaining highly technical matters such as artificial intelligence and machine learning. Although we believe this was a justified approach, our extraction of information might also have influenced the discussions.

We also tried to avoid letting our own views shine through too much in the discussions, but cannot be sure that this was completely avoided. As we progressed with the cafés certain technology choices were made in the project and we sought to be transparent about this and about the assumptions for the discussions in each café. Such topics of framing and transparency were discussed by the project team and with our ProjectSTEP group throughout the project. These framing issues are fundamental challenges in all involvement of participants with mild cognitive impairment – and indeed of participants without cognitive impairments – and is thus not an argument for or against dialogue cafés specifically.

## Conclusion

The dialogue cafés were carried out regularly for 3 years by a multidisciplinary research team with the intention of directing research and development and of engaging assisted living residents in technology discussions. The dialogue cafés served the overall aim of the Assisted Living Project in developing and evaluating technological solutions in the everyday life of assisted living residents. The study demonstrated that residents at an assisted living facility can contribute opinions about their needs for and perspectives on assistive technologies. This negates the view of older adults as frail and unable to participate. The study demonstrated that the engagement process was found to be positive, as expressed by the participants in the groups. It also succeeded in directing the research and technology development in the project. The dialogue cafés seemed to demonstrate a two-way learning process for both the researchers and the participants.

## Data Availability

The material in this project is written reports in Norwegian. They can be accessed in a de-identified format by contacting the first author.

## Abbreviations

ALP: Assisted Living Project
DC: Dialogue Cafés
RRI: Responsible Research and Innovation
TV: Television

## References

[1] WHO. World Report on Disability 2011. Geneva; 2011. https://www.who.int/disabilities/world_report/2011/report.pdf Retrieved 28092020

[2] OECD. Health at a Glance 2019. https://www.oecd-ilibrary.org/sites/4dd50c09-en/index.html?itemId=/content/publication/4dd50c09-en Retrieved 28092020.

[3] EU (2019). Ageing Europe: looking at the lives of older people in the EU. 2019 edition ed.

[4] Banner D, Bains M, Carroll S, Kandola DK, Rolfe DE, Wong C, Graham ID (2019). Patient and public engagement in integrated knowledge translation research: are we there yet?. Res Involvement Engagement.

[5] Meld.St. 15 (2017-2018) Leve hele livet : en kvalitetsreform for eldre. [A full life - all your life — A Quality Reform for Older Persons] Minestery of Health and Care Services on 04 May 2018. https://www.regjeringen.no/no/dokumenter/meld.-st.-15-20172018/id2599850/

[6] NOU. Innovasjon i omsorg [Innovative care]. Oslo; 2011. n

[7] Casagrande FD (2019). Sensor event and activity prediction using binary sensors in real homes with older adults [PhD.]. University of Oslo, Faculty of Mathematics and Natural Sciences, Department of Informatics Department of Mechanical, Electronics, and Chemical Engineering.

[8] Benjamin K, Potts HWW (2018). Digital transformation in government: lessons for digital health?.

[9] Crowe S, Fenton M, Hall M, Cowan K, Chalmers I. Erratum to: Patients’, clinicians’ and the research communities’ priorities for treatment research: there is an important mismatch. Res Involvement Engagement. 2015;1(1). 10.1186/s40900-015-0003-x PMC5598091 29062491

[10] Nygaard A, Halvorsrud L, Linnerud S, Grov EK, Bergland A (2019). The James Lind Alliance process approach: scoping review. BMJ Open.

[11] Kaisler RE, Missbach B (2020). Co-creating a patient and public involvement and engagement ‘how to’ guide for researchers. Res Involvement Engagement.

[12] Holthe T (2020). Assistive technology to support everyday living for home-dwelling older citizens with and without mild cognitive impairment and dementia [PhD].

[13] Romsland GI, Milosavljevic KL, Andreassen TA. Facilitating non-tokenistic user involvement in research. Res Involvement Engagement. 2019;5(1):18-32. 10.1186/s40900-019-0153-3. 10.1186/s40900-019-0153-3 PMC6551868 31183162

[14] Nymberg VM, Bolmsjö BB, Wolff M, Calling S, Gerward S, Sandberg M (2019). 'Having to learn this so late in our lives...' Swedish elderly patients' beliefs, experiences, attitudes and expectations of e-health in primary health care. Scand J Prim Health Care.

[15] Mihailidis A, Cockburn A, Longley C, Boger J (2008). The acceptability of home monitoring technology among community-dwelling older adults and baby boomers. Assist Technol.

[16] Holthe T, Halvorsrud L, Karterud D, Hoel KA, Lund A. Usability and acceptability of technology for community-dwelling older adults with mild cognitive impairment and dementia: a systematic literature review. Clinical Interv Aging. 2018;13:863. 10.2147/CIA.S154717 PMC5942395 29765211

[17] Larsen SM, Mortensen RF, Kristensen HK, Hounsgaard L, Larsen SM (2019). Older adults' perspectives on the process of becoming users of assistive technology: a qualitative systematic review and meta-synthesis. Disabil Rehabil Assist Technol.

[18] Gramstad A, Storli SL, Hamran T. Exploring the meaning of a new assistive technology device for older individuals. Disability and rehabilitation: Assistive technology. 2014;9(6):493-8. 10.3109/17483107.2014.921249 24839989

[19] Larsen SM, Hounsgaard L, Brandt Å, Kristensen HK (2019). “Becoming acquainted”: the process of incorporating assistive technology into occupations. J Occup Sci.

[20] Patomella A-H, Lovarini M, Lindqvist E, Kottorp A, Nygård L (2018). Technology use to improve everyday occupations in older persons with mild dementia or mild cognitive impairment: a scoping review. Br J Occup Ther.

[21] Sanders D, Scott P (2020). Literature review: technological interventions and their impact on quality of life for people living with dementia. BMJ Health Care Inform.

[22] Holroyd-Leduc J, Resin J, Ashley L, Barwich D, Elliott J, Huras P, Légaré F, Mahoney M, Maybee A, McNeil H, Pullman D, Sawatzky R, Stolee P, Muscedere J (2016). Giving voice to older adults living with frailty and their family caregivers: engagement of older adults living with frailty in research, health care decision making, and in health policy. Res Involvement Engagement.

[23] Domecq JP, Prutsky G, Elraiyah T, Wang Z, Nabhan M, Shippee N, et al. Patient engagement in research: a systematic review. Acta Vet Scand. 2014;14(1):89. 10.1186/1472-6963-14-89. 10.1186/1472-6963-14-89 PMC3938901 24568690

[24] Burrows A, Meller B, Craddock I, Hyland F, Gooberman-Hill R (2019). User involvement in digital health: working together to design smart home health technology. Health Expect.

[25] Prahalad CK, Ramaswamy V (2004). Co-creation experiences: the next practice in value creation. J Interact Mark.

[26] Leask CF, Sandlund M, Skelton DA, Altenburg TM, Cardon G, Chinapaw MJM (2019). Framework, principles and recommendations for utilising participatory methodologies in the co-creation and evaluation of public health interventions. Res Involvement Engagement.

[27] Zwass V (2010). Co-creation: toward a taxonomy and an integrated research perspective. Int J Electron Commer.

[28] Greenhalgh T, Jackson C, Shaw S, Janamian T (2016). Achieving research impact through co-creation in community-based health services: literature review and case study. Milbank Q.

[29] Askheim OP, Christensen K, Fluge S, Guldvik I. User participation in the Norwegian Welfare Context: an Analysis of Policy Discourses. 2017;46(3):583–601.

[30] Staley K, Barron D (2019). Learning as an outcome of involvement in research: what are the implications for practice, reporting and evaluation?. Res Involv Engagem.

[31] Wickson F, Forsberg EM (2015). Standardising responsibility? The significance of interstitial spaces. Sci Eng Ethics.

[32] Forsberg, EM. and Thorstensen, E. A Report from the Field: Doing RRI from Scratch in an Assisted Living Technology Research and Development Project. In: F. Ferri et al., Governance and Sustainability of Responsible Research and Innovation Processes, SpringerBriefs in Research and Innovation Governance, 2018; DOI: 10.1007/978-3-319-73105-6_3, pp. 19–26.

[33] ALP. Assisted Living Project 2019 [Available from: https://assistedlivingweb.wordpress.com/english/.

[34] Zouganeli E, Casagrande FD, Holthe T, Lund A, Halvorsrud L, Karterud D, Flakke-Johannessen A, Lovett H, Kjeang Mørk S, Strøm-Gundersen J, Thorstensen E, Norvoll R, ter Meulen R, Kennedy MR, Owen RJ, Ladikas M, Forsberg EM (2017). Responsible development of self-learning assisted living technology for older adults with mild cognitive impairment or dementia. Proceedings of ICT 4 Aging Well, Porto, Portugal.

[35] Holthe T, Casagrande FD, Halvorsrud L, Lund A. The assisted living project: a process evaluation of implementation of sensor technology in community assisted living. A feasibility study. Disabil Rehabil Assist Technol. 2018;1(1):29-36. 10.1080/17483107.2018.1513572 30318955

[36] Holthe T, Halvorsrud L, Lund A (2020). A critical occupational perspective on user engagement of older adults in an assisted living facility in technology research over three years. J Occup Sci.

[37] Thorstensen E (2020). Responsible Assessments. Frameworks for a Value-Based Governance of Assistive technologies. [PhD].

[38] Brown J, Isaacs D (2005). The world Café : shaping our futures through conversations that matter.

[39] Pålshaugen Ø (2014). Dialogues in innovation: interactive learning and interactive research as means for a context sensitive regional innovation policy.

[40] Engage 2020 (2014). Tools and instruments for a better societal engagement in "Horizon 2020" [Internet].

[41] MacFarlane A, Galvin R, O’Sullivan M, McInerney C, Meagher E, Burke D (2017). Participatory methods for research prioritization in primary care: an analysis of the world Café approach in Ireland and the USA. Fam Pract.

[42] Forsberg EM, Thorstensen E, Nielsen RO, Bakker E (2014). Assessments of emerging science and technologies: mapping the landscape. Sci Public Policy.

[43] Kennedy MR, Meulen ter R. Recommendations for involving people with dementia or mild cognitive impairment and their informal caregivers and relatives in the assisted living project. Bristol-Oslo: CEM Centre of Ethic and Medicine Oslo University College; 2016.

[44] Forskrift om tildeling av bolig i Omsorg+, Oslo kommune, Oslo [Regulation on 37 allocation of apartment in CARE+, Municipality of Oslo], LOV-1992-09-25-107-§6 C.F.R. 2011. https://lovdata.no/dokument/LTII/forskrift/2014-06-18-1026.

[45] Brinkmann S, Kvale S (2015). Interviews. Learning the craft of qualitative research interviewing.

[46] Casagrande FD, Tørresen J, Zouganeli E (2019). Predicting Sensor Events, Activities, and Time of Occurrence Using Binary Sensor Data From Homes With Older Adults.

[47] Crowe S, Adebajo A, Esmael H, Denegri S, Martin A, McAlister B (2020). ‘All hands-on deck’, working together to develop UK standards for public involvement in research. Res Involvement Engagement.

